# Calcium Sulfate and Calcium Carbonate Scaling of Thin-Film Composite Polyamide Reverse Osmosis Membranes with Surface-Tethered Polyacrylic Acid Chains

**DOI:** 10.3390/membranes12121287

**Published:** 2022-12-19

**Authors:** Yian Chen, Yoram Cohen

**Affiliations:** 1 Water Technology Research Center, Department of Chemical and Biomolecular Engineering, Henry Samueli School of Engineering and Applied Science, University of California, Los Angeles, CA 90095, USA; 2 Renewable Resources & Enabling Science Center, National Renewable Energy Laboratory, Golden, CO 80401, USA

**Keywords:** surface nano-structuring, brush layer, mineral scaling, gypsum, calcium carbonate, atmospheric pressure plasma-induced graft polymerization, membrane cleaning

## Abstract

The gypsum and calcite scaling propensities of the thin-film composite polyamide (PA-TFC) reverse osmosis (RO) membrane, modified with a tethered surface layer of polyacrylic acid (PAA) chains, was evaluated and compared to the scaling of selected commercial RO membranes. The tethered PAA layer was synthesized onto a commercial polyamide membrane (i.e., base-PA) via atmospheric pressure plasma-induced graft polymerization (APPIGP). The PAA nano-structured (SNS) base-PA membrane (SNS-PAA-PA) was scaled to a lesser degree, as quantified by a lower permeate flux decline and surface imaging, relative to the tested commercial membranes (Dow SW30, Toray SWRO, and BWRO). The cleaning of gypsum-scaled membranes with D.I. water flushing achieved 100% water permeability recovery for both the SNS-PAA-PA and Dow SW30 membranes, relative to 92–98% permeability restoration for the Toray membranes. The calcium carbonate scaling of SNS-PAA-PA membranes was also lower relative to the commercial membranes, but permeability recovery after D.I. water cleaning was somewhat lower (94%) but consistent with the level of surface scale coverage. In contrast, the calcite and gypsum-scaled membrane areas of the commercial membranes post-cleaning were significantly higher than for the SNS-PAA-PA membrane but with 100% permeability recovery, suggesting the potential for membrane damage when mineral scaling is severe.

## 1. Introduction

Reverse osmosis (RO) membrane desalination is now a mainstream technology utilized for the production of potable water from seawater (SW) and brackish water (BW) in water reuse applications and industrial water purification [1,2]. However, membrane fouling, and mineral scaling are the two critical process impediments that limit the attainable product water recovery and increase operational costs. Mineral scaling, which is the focus of the present work, is typically more problematic in inland water treatment and high-recovery desalination. Membrane scaling occurs when concentrations of sparingly soluble salts (e.g., CaCO_3_, CaSO_4_, BaSO_4_, SrSO_4_, and silica) exceed their solubility limits within the RO membrane feed channels [3,4,5,6]. Under such conditions, mineral scaling occurs due to (a) the precipitation/crystallization of sparingly soluble salts in the bulk solution, subsequent deposition onto the membrane surface, and continued crystal growth and/or (b) direct heterogeneous nucleation on the membrane surface and subsequent crystal growth. Membrane mineral scaling reduces membrane permeability and thus greater energy consumption is required for a given target permeate production, in addition to potential plant downtime, added cost of membrane cleaning, and shortening of membrane service life [3,7].

Current strategies for mitigating mineral scaling include (i) the removal of mineral scale precursor ions via feedwater pretreatment (e.g., chemical softening, ion exchange, nanofiltration); (ii) the inhibition of nucleation and mineral crystal growth via the dosing of antiscalants into the RO feedwater [8]; (iii) the adjustment of process operational conditions (e.g., reducing recovery to decrease the level of concentration polarization); (iv) periodic membrane permeate flushing; and (v) membrane chemical cleaning in place (CIP) [7]. In addition to the above, it is noted that since the early days of RO desalination, efforts have been devoted to developing membranes of higher resistance to fouling and scaling [9,10].

Previous studies on RO membrane mineral scaling have shown that membrane surface characteristics (e.g., surface charge, surface roughness/topography, and surface chemistry) can affect the rate of mineral crystal nucleation and growth, as well as scale surface adhesion [3,7,11,12]. Thus, different approaches for modifying the active layer of RO membranes have been explored with the aim of creating RO membranes of low scaling/fouling propensity, including, but not limited to: (i) physical coating with graphene oxide [13,14,15]; (ii) membrane surface topological microstructure patterning via imprint lithography [16,17,18]; (iii) the deposition of electrically conducting carbon nanotubes during polyamide (PA) active layer casting [19]; (iv) graft polymerization (“grafting-to”) [20]; and (v) polymer grafting (“grafting-from”) [13,21,22,23]. Of the above surface modification approaches, graft polymerization, used to form a surface layer of tethered hydrophilic polymers, has received significant attention given its ability to fine-tune membrane performance and reduce membrane fouling and mineral scaling propensities. For example, it was reported that PA thin-film composite (TFC) RO membranes that are modified via surface nano-structuring with a tethered layer of poly(methacrylic acid) and poly(acrylamide) could significantly reduce gypsum surface crystallization [21,22]. In another study, a zwitterionic polymer brush layer was synthesized onto a commercial TFC-PA RO membrane via atom transfer radical polymerization [23]. The modified RO membrane demonstrated superhydrophilicity (water contact angle <20°) and enhanced resistance to gypsum scaling [23]. RO membrane was also modified with the physical coating of GO nanomaterials followed by acrylic acid (AA) graft polymerization [13]. The modified RO membrane surface hydrophilicity increased, surface roughness decreased, and gypsum scaling tests demonstrated reduced flux decline [13]. It was suggested that the tethered hydrophilic polymer chains reduced mineral scaling owing to: (a) an increase in surface hydrophilicity which leads to a positive (repulsive) interfacial energy (for the scale crystals/membrane interface), thus reducing crystal adherence onto the membrane surface [23]; (b) screening of the underlying PA surfaces, hence reducing the adhesion of formed crystal nuclei onto the underlying membrane active layer [24]; and (c) reduction of heterogenous crystal nucleation rates (on the membrane surface) due to the partial mobility of the tethered chain segments [25]. Here, we note that it has been reported that there is likely to be a difference in surface adherence of different scalants (e.g., calcite and gypsum) [26]. Thus, one should expect that the rate and severity of membrane mineral scaling and the ease of membrane cleaning may also depend on the interaction between the specific mineral scalants and the membrane surface. It is noted that previous work, on the impact of TFC-PA membrane surface modification with tethered polyelectrolytes, focused on gypsum scaling but did not consider calcite which is also a commonly encountered membrane scalant. In the above studies, the impact of scaling was quantified via flux decline scaling tests, but fell short of assessing membrane cleanability and comparison with commercial membranes of similar permeability and rejection performance [13,21,22,27].

In the present study, we explore both gypsum and calcite membrane scaling, and subsequent cleaning (via simple D.I. water flush) for PA-TFC RO membranes with surface nano-structured (SNS) layers of tethered poly(acrylic acid) (PAA) chains. PAA was selected for surface modification given that previous studies have demonstrated that membrane surface nano-structuring with tethered PAA chains can effectively reduce the organic fouling of modified membranes [21,22,28]. Moreover, scale-up of the above PA membrane surface modification approach was shown to be feasible for the manufacturing of commercial-scale spiral-wound SWRO membrane elements [28]. The SNS membrane was synthesized by an established two-step process consisting of TFC-PA membrane surface activation via treatment with helium atmospheric pressure plasma (APP), followed by graft polymerization (GP) of acrylic acid (AA) to form a tethered layer of PAA chains (the surface modified membrane hereinafter termed SNS-PAA-PA). The membrane scaling of SNS-PAA-PA membranes with both calcium sulfate dihydrate (CaSO_4_·2H_2_O, known as gypsum) and calcium carbonate (CaCO_3_ as calcite) was evaluated (in flux decline scaling tests) relative to that of selected commercial membranes. In addition, membrane cleaning via mere water flushing was assessed with respect to scale removal and permeability recovery.

## 2. Materials and Methods

### 2.1. Materials

A commercial PA-TFC BWRO membrane (obtained as flat sheets; 73AC, Toray Membrane USA Inc., Poway, CA, USA) with sufficiently high water permeability coefficient was selected as the base membrane to produce a poly(acrylic acid) (PAA) surface nano-structured (SNS) PA membrane. Two commercial membranes, Toray SWRO (82V, Toray Membrane USA Inc., Poway, CA, USA) and Dow SW30 (SW30HR, Dow Co., Midland, MI, USA), were selected for performance comparison, in scaling tests, with the SNS-PAA-PA membrane (Table 1).

Surface nano-structuring of the base membrane was achieved via acrylic acid (99%, Sigma-Aldrich, St. Louis, MO, USA) graft polymerization, initiated by atmospheric pressure He plasma surface activation. A 0.1 N NaOH aqueous solution, prepared in D.I. water using 50% *w*/*w* sodium hydroxide solution (Fisher Scientific, Chino, CA, USA), served to adjust the monomer solution pH. Helium (99.999%) and oxygen (99.999%) gases (Airgas, Los Angeles, CA, USA) were the atmospheric pressure plasma treatment sources. Nitrogen (99%) gas (Airgas, Los Angeles, CA, USA) was used for membrane surface drying prior to plasma surface activation and monomer solution degassing during graft polymerization.

Model solutions for the membrane scaling tests were prepared using calcium chloride dihydrate (CaCl_2_, Certified ACS, Fisher Scientific, Chino, CA, USA), sodium sulfate (Na_2_SO_4_, Certified ACS, Fisher Scientific, Chino, CA, USA), and sodium bicarbonate (NaHCO_3_, ≥99.5%, Acros Organics, Freehold, NJ, USA), along with sodium chloride (NaCl, ≥99.0%, Fisher Scientific, Chino, CA, USA) that was used to adjust the solution’s salinity.

### 2.2. Atmospheric-Pressure-Induced Graft Polymerization (APPIGP)

Polyamide membrane surface nano-structuring (permeability and salt rejection) was accomplished following a previously established protocol [28] to achieve SNS-PAA-PA membrane salt selectivity proximal to the performance of the Dow SW30 membrane. Prior to membrane surface plasma treatment, membrane coupons (with active areas of 42 cm^2^) were extracted from a flat sheet of the base-PA membrane, rinsed with D.I. water, and blow-dried with compressed nitrogen. The base-PA membrane active polyamide surface was activated by exposing the surface to He atmospheric pressure plasma (APP) via an impinging stream plasma source (Atomflo™ 500; Surfx Technologies Inc., Redondo Beach, CA, USA). Plasma was generated at 150W RF power with a helium flow rate of 45 L/min. The plasma source head was translated over the base-PA membrane via a scanning robot at a speed of 100 mm/s, whereby plasma surface treatment was accomplished via 2 sequential plasma scans (N) at a source-surface separation (PSS) distance of 10 mm. The above step served to generate free radicals that were converted into peroxide groups (–O-O or –O-O-H) upon exposure to ambient air [29]. Subsequently, the plasma-treated membrane samples were immersed in 250 mL glass reaction vessels containing the aqueous AA monomer solutions to initiate surface free-radical polymerization. The reaction vessels were placed in a constant water bath and the reaction proceeded at 70 °C for 60 min. Thermal decomposition of the surface peroxide groups served to initiate acrylic acid free-radical graft polymerization [29]. Nitrogen was bubbled into the monomer solution (via a perforated tube) to both scavenge dissolved oxygen that could inhibit the graft polymerization reaction and facilitate mixing [30]. The initial monomer concentration ([M]_o_) was 21 vol% and the solution pH was adjusted to 6 using 0.1 N aqueous NaOH solution. It is emphasized that the above graft polymerization approach results in surface-tethered PAA chains are covalently anchored to the membrane surface as confirmed in previous studies [29,31,32]. After graft polymerization, the membrane samples were thoroughly rinsed and stored in D.I water prior to membrane characterization and the mineral scaling tests. It is noted that the resulting SNS-PAA-PA membranes had a water permeability of 1.69 ± 0.18 L·m^−1^·h^−1^·bar^−1^ and a nominal salt (NaCl) rejection of 99.3 ± 0.1% [28]. The above membrane performance is within the acceptable range for seawater desalination membranes [28,33].

### 2.3. Surface Scanning Images

Images of membrane surfaces [28,34] were obtained using a scanning electron microscope (SEM) (Zeiss Supra VP40, Carl Zaiss AG, Oberkochen, Germany). Prior to SEM surface characterization, the membrane samples were rinsed with D.I. water and dried in a vacuum oven at 40 °C for 24 h. The fully dried samples were sputter-coated (Hummer^®^ 6.6 Sputter Coater, Anatech USA, Sparks, NV, USA) for 3 min to form a thin film of gold (Au) to prevent surface charging during SEM characterization. SEM scanning was carried out with an accelerating voltage of 10 keV and a spot size of 100–10,000 nm. All images were obtained at a working distance of 5 mm and a magnification of 15,000.

### 2.4. Membrane Performance Characterization

#### 2.4.1. Permeability

Membrane water permeability was determined using a laboratory plate-and-frame RO (PFRO) membrane recirculation unit. The PFRO system consisted of a rectangular flow cell (CF042D; Sterlitech Corp., Kent, WA, USA) accommodating an active membrane area of 42 cm^2^. A positive displacement pump (Hydra-Cell; Wanner Engineering Inc., Minneapolis, MN, USA) delivered the feed solution from a 15 L feed tank. Feed solution temperature was maintained at 20.0 ± 0.2 °C using the refrigerated bath circulator (RTE-221, NESLAB Instruments Inc., Newington, NH, USA), monitored with a temperature probe (Go!Temp; Vernier Software & Technology, Beaverton, OR, USA). Transmembrane pressure was adjusted with a back-pressure valve (MCJ-050AB-3-1335G4Y; Hanbay Inc., Virginia Beach, VA, USA) at the RO unit concentrate exit, and monitored via a pressure transmitter (Model A-10; WIKA Instrument LP, Lawrenceville, GA, USA). The concentrate and permeate volumetric flow rates were monitored with a liquid flow sensor (Model 101-7; McMillan, Georgetown, TX, USA) and a digital liquid flow meter (Model 5025000; GJC Instruments Ltd., Tattenhall Chester, UK), respectively.

Prior to determining the membrane water permeability coefficient, the membrane coupons were compacted, under D.I. water flow (channel crossflow velocity of 49 cm/s) at a transmembrane pressure (Δ*P*) of 58.6 bar (~850 psi) at ~20 °C until the permeate flux stabilized (typically within 24 h). Membrane D.I. water permeability was determined from permeate flux measurements over a transmembrane pressure range of 34.5–58.6 bar (500–850 psi). The membrane water permeability coefficient, $L_{p}=J_{v}/∆P$, was then determined from the slope of the permeate water flux, $J_{v}=Q_{p}/A$ (where $Q_{p}$ is the permeate flow rate and *A* is the active membrane area) versus the transmembrane pressure.

#### 2.4.2. Mineral Scaling Tests

Prior to the mineral scaling tests, the membranes were compacted, and the clean membrane water permeability coefficient was determined with D.I. water (Section 2.4.1). Membrane mineral scaling was assessed using calcium sulfate and calcium carbonate model solutions (Table 2). The model solutions were prepared to yield saturation levels, with respect to the calcite and gypsum, that mimicked those of brackish groundwater in the agricultural area of the California Panoche Drainage District [35]. The calcium sulfate model solution was prepared by dissolving CaCl_2_, Na_2_SO_4_, and NaCl salts in D.I. water. The calcium carbonate scaling solution was prepared by dissolving CaCl_2_, NaHCO_3_, and NaCl in D.I. water. The salinity of both solutions was 2611 mg/L of total dissolved solids (TDSs).

Saturation levels with respect to gypsum and calcite for the above scaling solutions were quantified in terms of the saturation index, defined as $SI_{x}=IAP/K_{sp,x}$, where *IAP* is the ion activity product and $K_{sp,x}$ is the solubility product for the mineral salt x (e.g., where *x* = *c* is CaCO_3_ (as calcite) and *x* = *g* is gypsum). The gypsum and calcite saturation indices for the model scaling solutions were ~1 and 6.3, respectively. At the membrane surface, the saturation indices for gypsum and calcite were assessed based on the estimated solute concentrations at the membrane surface, i.e., $C_{m}=CP\times C_{b}$, where $C_{b}$ is the bulk solute concentration and *CP* is the concentration polarization modulus estimated as detailed in a previous study [28].

The scaling experiments were conducted in the mode of total recycle in which the permeate and retentate streams were continuously recirculated back to the feed reservoir. The scaling tests were conducted over a 24 h period at ~20 °C, at a crossflow velocity of 49 cm/s and an initial permeate flux of 39.4 L·m^−2^·h^−1^. This high crossflow velocity was selected such that the concentration polarization modus, *CP*, at the membrane surface could be kept as low as 1.3 [28]. At the above operating conditions, the average saturation indexes of gypsum (*SI_g_*) and calcite (*SI_c_*) at the membrane surface were 1.7 and 10.6, respectively. After each scaling test, D.I. water was circulated (for 30 min) through the PFRO system (~20 °C), at a crossflow velocity of 49 cm/s and a transmembrane pressure of 21.4 bar (~310 psi). Following the above membrane cleaning with D.I water, the water permeability coefficient ($L_{p}^{'}$) was again determined with D.I. water as per the protocol described in Section 2.4.1. The membrane cleaning efficacy was then quantified in terms of the permeability recovery, defined as the permeability recovery $=\left(L_{p}^{'}/L_{p}\right)\times 100\%$.

## 3. Results and Discussion

### 3.1. Membrane Scaling

**Figure 1:**
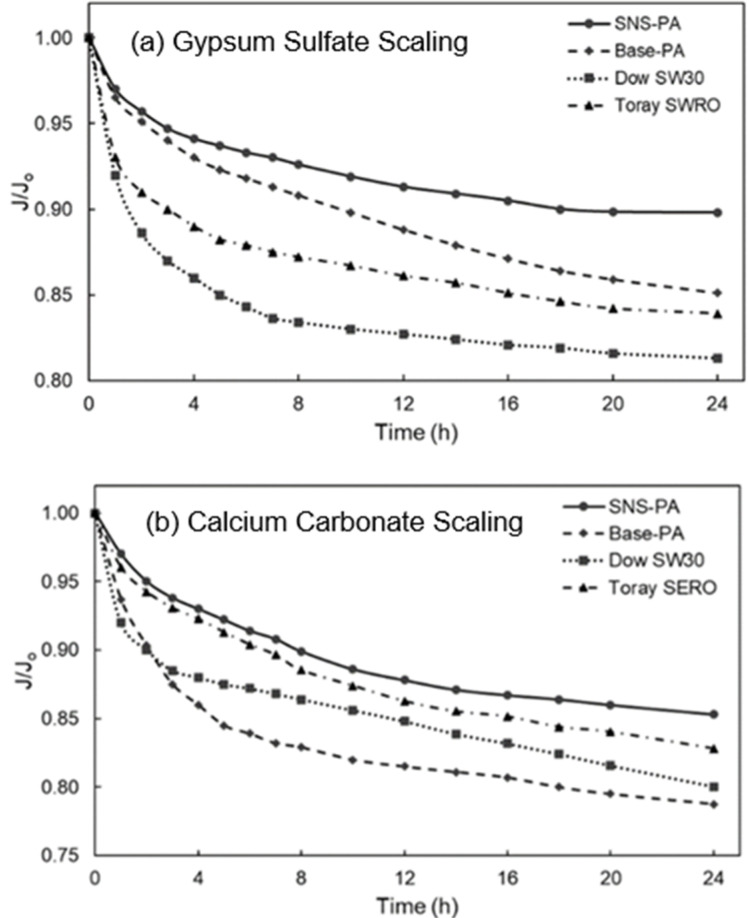
Flux decline for the SNS-PAA-PA membrane after 24 h calcium sulfate scaling (**a**) and calcium carbonate scaling (**b**) tests, also showing the corresponding flux decline curves for the commercial SW30, Toray SWRO, and base-PA membranes. (Scaling tests conditions: Calcium sulfate feed solution pH of 5.7, membrane surface saturation index of gypsum *SI_g_* = 1.7; calcium carbonate feed solution pH of 7.9, membrane surface saturation index of calcite *SI_c_* = 10.6.)

The calcium sulfate and calcium carbonate scaling tests (Section 2.4.2) led to mineral scaling that resulted in rapid permeate flux decline (Figure 1) for both SNS-PAA-PA and the three commercial RO membranes (i.e., Dow-SW30, Toray SWRO, and base-PA (Toray 73AC)). The SNS-PAA-PA membrane demonstrated lower flux decline relative to the commercial BWRO (i.e., base-PA) and SWRO (i.e., Dow SW30 and Toray SWRO) membranes in both the calcium sulfate and calcium carbonate scaling tests. At the end of the 24 h calcium sulfate scaling tests, the SNS-PAA-PA membrane demonstrated a permeate flux decline of 10% relative to 19%, 16%, and 15% for the commercial Dow-SW30, Toray SWRO, and base-PA membranes (Figure 1, Table 3), respectively. Similarly, the calcium carbonate scaling tests revealed a permeate flux decline (at t = 24 h) of 15% for the SNS-PAA-PA membrane relative to 20%, 17%, and 21% for the Dow-SW30, Toray SWRO, and base-PA membranes, respectively (Figure 1).

**Figure 2:**
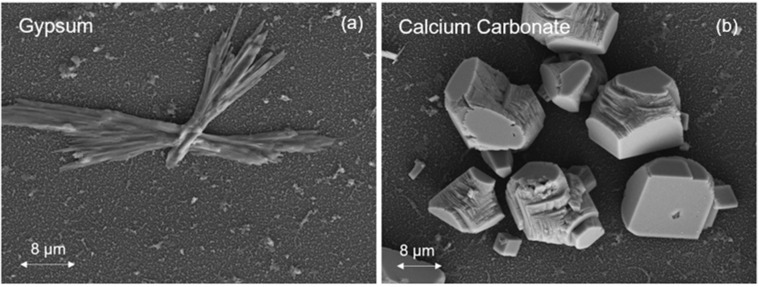
SEM top-view images of the gypsum (**a**) and calcite (**b**) scale on the SNS-PAA-PA membrane surface (at the end of 24 h scaling tests). (Saturation indices at the membrane surface: *SI_g,m_* = 1.7 and *SI_c.m_* = 10.6.).

**Figure 3:**
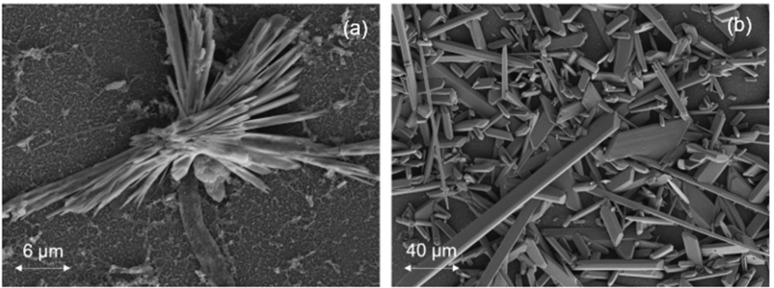
SEM images of scaled membrane surfaces revealing different gypsum crystal structures: (**a**) rosette arrangement (on Dow SW30 membrane) consisting of gypsum platelets extending from a core region and (**b**) individual needle-shaped platelet structures (on Toray SWRO membrane) (images are at the end of the 24 h scaling tests).

Given that the calcium carbonate solution was supersaturated (*SI_c_* = 6.3, Table 2) and the calcium sulfate solution was just saturated (*SI_g_* = 1.0, Table 2), crystallization of the respective mineral salts was expected to occur both in the bulk of the solution and directly on the membrane surfaces [6,12]. Crystal scales (due to both surface crystallization and the deposition of bulk-formed crystals onto the membrane surface) can block the membrane surface, thereby reducing membrane water permeability [12]. Gypsum crystals of platelet structure (i.e., orthorhombic or hexagonal prismatic) were observed, as illustrated in Figure 2; such crystal structures have been reported to occur primarily due to deposition of bulk-formed crystals [36,37]. Gypsum crystals also appeared in the form of rosette arrangements (consisting of gypsum platelets emanating from a core region; Figure 3) which have been shown to form by surface crystallization [7,38,39]. Calcium carbonate scale crystals on the membrane surface (Figure 2) was were of the stable anhydrous rhombohedral calcite polymorph [12,40].

**Figure 4:**
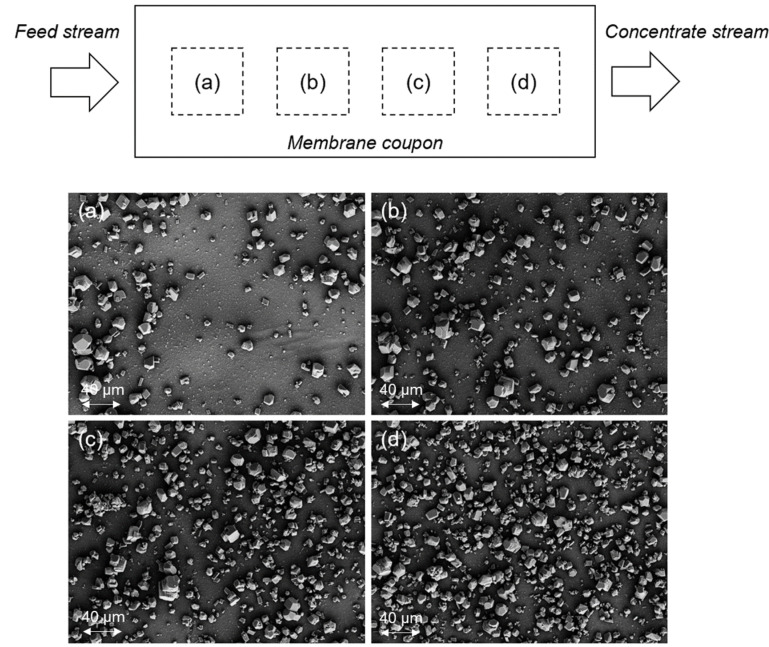
SEM top-view images of Toray SWRO membrane after 24 h calcite scaling tests at increasing axial positions (**a**–**d**) from the channel entrance. The dashed squares a to d are 1 × 1 cm areas extracted from membrane coupon for SEM characterization. (Note: membrane surface calcite scale coverage at positions (a), (b), (c), and (d) were 16.3%, 29.7%, 42.4%, and 54.9%, respectively. The images in (**a**–**d**) were taken at distances of 2, 3.7, 5.4, and 7.1 cm from the RO channel entrance, respectively.)

**Figure A1:**
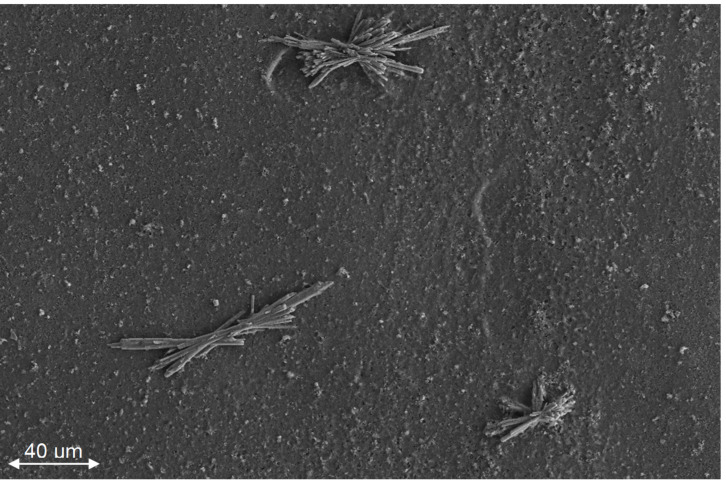
SEM surface image of the gypsum-scaled SNS-PAA-PA membrane. (Scaling test conditions: 24 h filtration with gypsum feed solution pH of 5.7, membrane surface saturation index of gypsum *SI_g,m_* = 1.3).

The deposition and growth of mineral salt crystals along the membrane surface were nonuniform (Figure 4). As water recovery increased along the membrane channel (i.e., from channel inlet to outlet in the axial flow direction), concentration polarization increased leading to rising concentrations of the scale precursor ions. As a consequence, the nucleation rate and crystal diffusional growth both increased with rising mineral salt supersaturation [41]. Therefore, the crystal number density on the membrane surface should increase axially as one progresses from the entrance toward the exit region of the RO channel (Figure 4), consistent with previous studies [38,42,43,44]. Indeed, the membrane surface calcite coverage increased from 16.3% to 29.7%, 42.4%, and 54.9%, for positions (a), (b), (c), and (d) in Figure 4, respectively. The above trend was more evident for the scaling tests in which the calcite saturation index was 10.6 at the membrane surface, relative to the lower 1.7 value for gypsum (Figure A1, Appendix A).

### 3.2. Membrane Cleaning and Water Permeability Recovery

**Figure A2:**
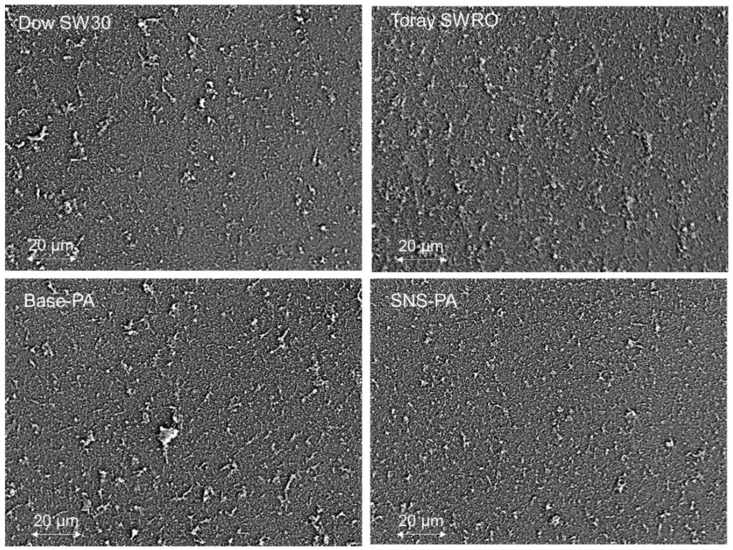
SEM top-view images of SNS-PA, Dow SW30, Toray SWRO, and base-PA membranes after 24 h gypsum scaling tests, followed by 30 min D.I. water flushing. (Scaling test conditions: 24 h filtration with gypsum feed solution (pH of 5.7) and a membrane surface saturation index of gypsum *SI_g,m_* = 1.3).

The cleaning of gypsum-scaled SNS-PAA-PA and Dow SW30 membranes with D.I. water flushing (Section 2.4.2) resulted in 100% water permeability recovery (Table 3). Water Permeability restoration for the base-PA and Toray SWRO membranes was somewhat lower, i.e., ~92% and ~98%, respectively. The above levels of water permeability recovery for the gypsum-scaled membrane, upon cleaning with D.I. water, are consistent with the post-cleaning SEM images (Figure A2, Appendix A) and with previous studies [45,46]. Restoring water permeability of the gypsum-scaled membrane was likely to have been achieved via (i) dissolution of surface gypsum crystals [47], given the measurable gypsum aqueous solubility (i.e., 2.53 g/L at 20 °C [48]), and (ii) shear removal of loosely surface bound/attached mineral crystals. It was postulated that the lower level of gypsum surface scaling and the higher cleaning efficacy of the gypsum-scaled SNS-PAA-PA membrane, relative to the commercial RO membranes, are due to the surface screening of the membrane active PA layer via the tethered hydrophilic PAA brush layer and a reduction in adhesion between the mineral crystals and the underlying membrane surface. Upon cleaning, the tethered PAA chains which are in a collapsed configuration at the high ionic strength of the scaling solution (80.81 mM) (due to charge screening effect [49,50,51]), swell when exposed to D.I. water (due to the electrostatic repulsion among the charged PAA chain segments). The above chain swelling is postulated to promote the detachment of scale crystals [29,52,53,54,55,56,57,58,59] from the PAA-tethered surface layer.

**Figure 5:**
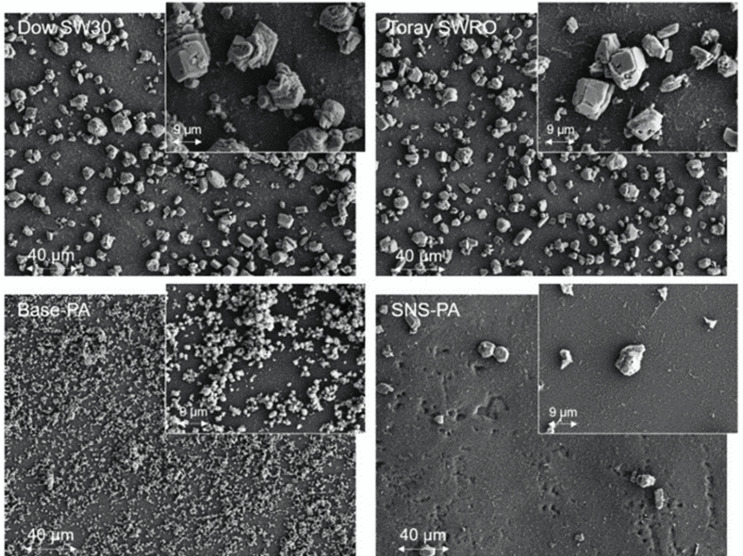
SEM top-view images of the SNS-PAA-PA and commercial membranes (Dow SW30, Toray SWRO, and base-PA) after D.I. water cleaning of membranes that were scaled with calcium carbonate over a 24 h scaling test. Calcite surface scale coverage (%) in the imaged zones: 40.5%, 59.9%, 57.8%, and 4.3% for the Dow SW30, Toray SWRO, base-PA, and SNS-PAA-PA membranes, respectively. (Note: all images were taken at a distance of 5.4 cm from the RO channel entrance (equivalent to image Figure 4c).

**Figure A3:**
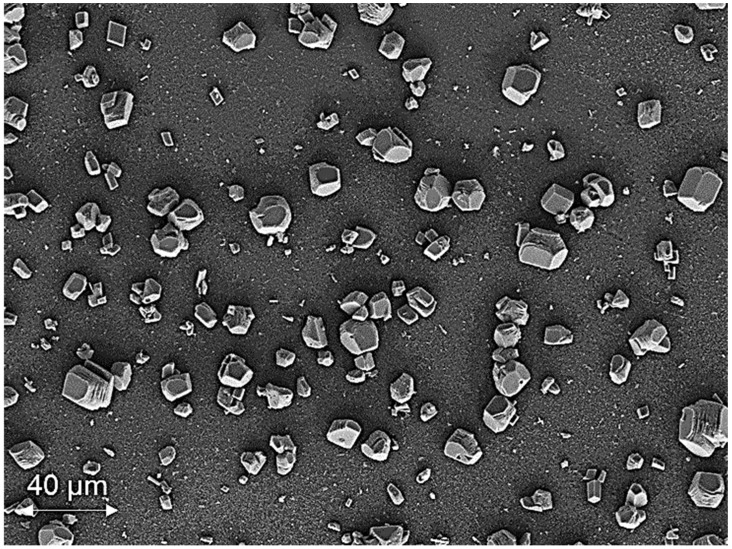
SEM top-view image of SNS-PA membrane after 24 h calcium carbonate scaling test (crystal surface coverage ~25%).

Permeability recovery for the calcite-scaled SNS-PAA-PA membrane, upon D.I. water cleaning, was somewhat lower (94%) than for the gypsum-scaled membrane. Consistent with the above, SEM images of the SNS-PAA-PA membrane reveal a residual scaled calcite area of ~4% (in the imaged membrane; Figure 5) after cleaning, relative to the scaled membrane area of 25% (Figure A3, Appendix A) at the end of the scaling test. Here, we note that the low calcite solubility in water (~0.015 g/L [60] at 20 °C and the D.I. water pH of ~6.0) and observed reduced calcite surface number density after cleaning (Figure 5) suggest that membrane cleaning likely to have occurred due to shear removal of loosely surface-adhered calcite crystals that deposited onto the membrane surface from the bulk solution. In contrast with the above, complete permeability recovery was attained for the calcite-scaled commercial membranes upon D.I. water cleaning (Table 3). However, the residual-scaled area after D.I. water cleaning was greater for the commercial membranes (~40.5–59.9%) relative to the scaled SNS membrane. The above suggests that the active PA layer of the commercial membranes may have been damaged to some degree which could have accounted for the increased recovery observed after membrane cleaning. Here, we note that mineral crystals can damage the membrane active PA layer, as suggested in previous studies [61,62,63,64]; thus, membrane cleaning at the early stages of membrane scaling may be preferable in order to avoid membrane damage.

The formation of the mineral scale is governed by the rate of nucleation on the membrane surface and in the bulk solution, crystal adhesion onto the membrane surface, and the rate of crystal growth which is impacted by the level of solution supersaturation and local hydrodynamics. Membrane surface properties (e.g., surface hydrophilicity and surface roughness) should thus be expected to have a measurable impact on membrane surface mineral scale formation [7,12,65,66,67]. Hence, as demonstrated in the present study, there are significant differences in the scaling propensities of the different membranes, as reflected by the size distribution and mineral crystal adherence. For example, the base-PA membrane had a captive bubble (CB) contact angle of 43.9° which was ~5–117% higher compared to the other three tested membranes [28]. The above implies greater membrane surface hydrophilicity, and thus a lower surface energy barrier for heterogeneous nucleation [7], as well as a greater mineral scaling propensity (and hence membrane water permeability decline). It is interesting to note that the size and apparent morphology of the residual surface crystals on the membranes, post-D.I. water flushing, varied considerably among the different membranes. For example, calcite crystals formed on the base-PA membrane were in the size range of (~1 μm) relative to ~3–17 μm for crystals on the Dow SW30, Toray SWRO, and SNS-PAA-PA membranes. Furthermore, the crystal surface number density was ~100–150 times greater on the base-PA membrane relative to the other three tested membranes (Figure 5). Gypsum crystals on the SNS-PAA-PA membrane were in the size range of 4–10 μm, while the crystal surface number density (1.3 × 10^5^/cm^2^) was a factor of 7–800 lower (relative to the commercial membranes). In conclusion, membrane mineral scaling and D.I water cleaning revealed that membrane scaling strongly depends on the membrane type and, as previous studies have shown, surface properties (e.g., topography, charge, hydrophilicity, and chemistry) [7,12,65,66,67].

## 4. Conclusions

The scaling propensity of the PA-TFC RO membrane modified with a surface-tethered polyacrylic acid (PAA) brush layer was evaluated and compared to commercial RO membranes. The PAA layer was surface-nano-structured (SNS) onto a commercial polyamide (PA) brackish water reverse osmosis (BWRO) membrane (i.e., base-PA) via atmospheric pressure plasma-induced graft polymerization (APPIGP). The SNS-PAA-PA membrane demonstrated lower scaling propensity, relative to commercial membranes (Dow SW30, and Toray SWRO and BWRO membranes), as indicated by its lowest permeate flux decline. The lower scaling propensity of the SNS-PAA-PA membrane can be attributed to screening of underlying PA surfaces by the tethered PAA layer and possibly lower nucleation rates owing to partial Brownian motion of segments of the tethered PAA chains. Simple membrane cleaning via D.I. water flushing of the gypsum-scaled membranes provided complete water permeability recovery for the SNS-PAA-PA and Dow SW30 membranes, relative to 92–98% permeability recovery for the commercial Toray membranes. Calcite scaling was similarly lowest for the SNS-PAA-PA membrane and consistent with the percent-scaled area, as revealed by SEM imaging of the scaled membrane. Upon D.I. water cleaning, water permeability recovery for the calcite-scaled commercial membranes was complete; however, SEM images of the cleaned membranes revealed significant residual-scaled areas, suggesting that the permeability loss should have been significantly higher. Likewise, the residual-scaled area for the gypsum-scaled commercial membranes (post-D.I. membrane cleaning) was significantly higher than suggested by the observed membrane water permeability loss. The above results suggest the possibility of membrane damage when mineral scaling is severe. Thus, there is merit in further investigation to quantify the impact of the severity of mineral scaling and membrane cleaning protocols in relation to membrane performance restoration and hence membrane longevity.

## Figures and Tables

**Table 1:** Separation performance of the SNS-PAA-PA and commercial membranes ^(a)^.

Membrane ^(b)^	Permeability ^(c)^ (L·m^−1^·h^−1^·bar^−1^)	Rejection ^(d)^ (%)	
		Observed	Intrinsic
SNS-PAA-PA	1.69	99.3	99.5
Toray SWRO	1.33	99.0	99.2
Base-PA	2.91	99.0	99.3
Dow SW30	1.60	89.9	99.2

^(a)^ Based on raw data reported in [28]. ^(b)^ SNS-PAA-PA, Toray SWRO, and base-PA membrane coupons were extracted from full-size flat membrane sheets. Dow SW30 membrane coupon was extracted from a membrane sheet removed from a spiral wound element (Dow Filmtec SW30-2514). ^(c)^ Membrane water permeability coefficients were determined for D.I. water after 24 h compaction at 850 psi. ^(d)^ Membrane salt rejection was determined using aqueous 35,000 mg/NaCl solution. Both water permeability and salt rejection following the protocol are described in [28].

**Table 2:** Ionic compositions of calcium sulfate and calcium carbonate model solutions.

Ions	Model Solution Composition ^(a)^	
	Calcium Sulfate	Calcium Carbonate
Ca^2+^	11.29 mM	11.29 mM
Na^+^	127.95 mM	160.33 mM
Cl^−^	33.67 mM	178.89 mM
SO_4_^2−^	58.43 mM	-
HCO_3_^−^	-	4.02 mM
pH	5.7	7.9
TDS	2611 mg/L	2611 mg/L
Saturation index in solution ^(b)^	*SI_g_* = 1.0	*SI_c_* = 6.3
Saturation index at membrane surface ^(c)^	*SI_g,m_* = 1.7	*SI_c,m_* = 10.6

^(a)^ The model solutions were prepared without pH adjustment. ^(b)^ *SI_g_* and *SI_c_*—gypsum and calcite saturation indices, respectively. ^(c)^ *SI_g,m_* and *SI_c,m_*—gypsum and calcite saturation indices at the membrane surface.

**Table 3:** Summary of membrane gypsum and calcite scaling and cleaning tests ^(a)^.

Membrane	Scalant	*J/J_o_* ^(b)^	*L_p_/L_p,o_* ^(c)^
SNS-PAA-PA	Gypsum	0.89	1.00
Dow SW30	Gypsum	0.81	1.00
Toray SWRO	Gypsum	0.84	0.98
Base-PA	Gypsum	0.85	0.92
SNS-PAA-PA	Calcite	0.85	0.94
Dow SW30	Calcite	0.80	1.00
Toray SWRO	Calcite	0.83	1.00
Base-PA	Calcite	0.79	1.00

^(a)^ Membrane flux decline tests (with membrane coupons) with results given at the end of 24 h gypsum and calcite scaling tests and water permeability recovery after 30 min D.I. water flush. The procedure is described in Section 2.4.2. ^(b)^ *J/J_o_*—ratio of membrane permeate flux at the end of scaling test/initial clean membrane permeate flux. ^(c)^ *L_p_/L_p,o_*—ratio of membrane water permeability coefficient after cleaning/initial permeability coefficient of the pristine membrane.

## Data Availability

The data presented in this study are available upon request from the corresponding author.

## References

[1] Malaeb L., Ayoub G.M. (2011). Reverse osmosis technology for water treatment: State of the art review. Desalination.

[2] Garud R., Kore S., Kore V., Kulkarni G. (2011). A Short Review on Process and Applications of Reverse Osmosis. Univers. J. Environ. Res. Technol..

[3] Matin A., Rahman F., Shafi H.Z., Zubair S.M. (2019). Scaling of reverse osmosis membranes used in water desalination: Phenomena, impact, and control; future directions. Desalination.

[4] Zhao S., Zou L., Mulcahy D. (2012). Brackish water desalination by a hybrid forward osmosis–nanofiltration system using divalent draw solute. Desalination.

[5] Hegab H.M., Zou L. (2015). Graphene oxide-assisted membranes: Fabrication and potential applications in desalination and water purification. J. Membr. Sci..

[6] Rahardianto A., Shih W.-Y., Lee R.-W., Cohen Y. (2006). Diagnostic characterization of gypsum scale formation and control in RO membrane desalination of brackish water. J. Membr. Sci..

[7] Tong T., Wallace A.F., Zhao S., Wang Z. (2019). Mineral scaling in membrane desalination: Mechanisms, mitigation strategies, and feasibility of scaling-resistant membranes. J. Membr. Sci..

[8] Hasson D., Shemer H., Brook I., Zaslavschi I., Semiat R., Bartels C., Wilf M. (2011). Scaling propensity of seawater in RO boron removal processes. J. Membr. Sci..

[9] Rana D., Matsuura T. (2010). Surface Modifications for Antifouling Membranes. Chem. Rev..

[10] Asadollahi M., Bastani D., Musavi S.A. (2017). Enhancement of surface properties and performance of reverse osmosis membranes after surface modification: A review. Desalination.

[11] Karanikola V., Boo C., Rolf J., Elimelech M. (2018). Engineered Slippery Surface to Mitigate Gypsum Scaling in Membrane Distillation for Treatment of Hypersaline Industrial Wastewaters. Environ. Sci. Technol..

[12] Kang N.W., Lee S., Kim D., Hong S., Kweon J.H. (2011). Analyses of calcium carbonate scale deposition on four RO membranes under a seawater desalination condition. Water Sci. Technol..

[13] Ashfaq M.Y., Al-Ghouti M.A., Zouari N. (2020). Functionalization of reverse osmosis membrane with graphene oxide and polyacrylic acid to control biofouling and mineral scaling. Sci. Total. Environ..

[14] Ansari A., Peña-Bahamonde J., Wang M., Shaffer D.L., Hu Y., Rodrigues D.F. (2021). Polyacrylic acid-brushes tethered to graphene oxide membrane coating for scaling and biofouling mitigation on reverse osmosis membranes. J. Membr. Sci..

[15] Cao B., Ansari A., Yi X., Rodrigues D.F., Hu Y. (2018). Gypsum scale formation on graphene oxide modified reverse osmosis membrane. J. Membr. Sci..

[16] Maruf S.H., Greenberg A.R., Pellegrino J., Ding Y. (2014). Fabrication and characterization of a surface-patterned thin film composite membrane. J. Membr. Sci..

[17] Choi W., Lee C., Lee D., Won Y.J., Lee G.W., Shin M.G., Chun B., Kim T.-S., Park H.-D., Jung H.W. (2018). Sharkskin-mimetic desalination membranes with ultralow biofouling. J. Mater. Chem. A.

[18] Wiese M., Nir O., Wypysek D., Pokern L., Wessling M. (2018). Fouling minimization at membranes having a 3D surface topology with microgels as soft model colloids. J. Membr. Sci..

[19] Duan W., Dudchenko A., Mende E., Flyer C., Zhu X., Jassby D. (2014). Electrochemical mineral scale prevention and removal on electrically conducting carbon nanotube—polyamide reverse osmosis membranes. Environ. Sci. Process. Impacts.

[20] Ray J.R., Wong W., Jun Y.-S. (2017). Antiscaling efficacy of CaCO_3_and CaSO_4_on polyethylene glycol (PEG)-modified reverse osmosis membranes in the presence of humic acid: Interplay of membrane surface properties and water chemistry. Phys. Chem. Chem. Phys..

[21] Kim M.-M., Lin N.H., Lewis G.T., Cohen Y. (2010). Surface nano-structuring of reverse osmosis membranes via atmospheric pressure plasma-induced graft polymerization for reduction of mineral scaling propensity. J. Membr. Sci..

[22] Lin N.H., Kim M.-M., Lewis G.T., Cohen Y. (2010). Polymer surface nano-structuring of reverse osmosis membranes for fouling resistance and improved flux performance. J. Mater. Chem..

[23] Jaramillo H., Boo C., Hashmi S.M., Elimelech M. (2020). Zwitterionic coating on thin-film composite membranes to delay gypsum scaling in reverse osmosis. J. Membr. Sci..

[24] Kang G.-D., Cao Y.-M. (2012). Development of antifouling reverse osmosis membranes for water treatment: A review. Water Res..

[25] Chen Y., Rovira-Bru M., Giralt F., Cohen Y. (2021). Hydraulic Resistance and Protein Fouling Resistance of a Zirconia Membrane with a Tethered PVP Layer. Water.

[26] Sheikholeslami R., Ong H. (2003). Kinetics and thermodynamics of calcium carbonate and calcium sulfate at salinities up to 1.5 M. Desalination.

[27] Yin Y., Kalam S., Livingston J.L., Minjarez R., Lee J., Lin S., Tong T. (2021). The use of anti-scalants in gypsum scaling mitigation: Comparison with membrane surface modification and efficiency in combined reverse osmosis and membrane distillation. J. Membr. Sci..

[28] Chen Y., Kim S., Kim Y., Walker J.S., Wolfe T., Coleman K., Cohen Y. (2022). Scale up of polyamide reverse osmosis membranes surface modification with tethered poly(acrylic acid) for fabrication of low fouling spiral-wound elements. Desalination.

[29] Chen Y., Zhang J., Cohen Y. (2022). Fouling resistant and performance tunable ultrafiltration membranes via surface graft polymerization induced by atmospheric pressure air plasma. Sep. Purif. Technol..

[30] Pourjavadi A., Zohuriaan-Mehr M.J. (2002). Modification of carbohydrate polymers via grafting in air. 1. ceric-induced synthesis of starch-g-polyacrylonitrile in presence and absence of oxygen. Starch-Stärke.

[31] Kim S., Chen Y., Yoram C. (2022). Surface Modified Reverse Osmosis Membranes. The World Scientific Reference of Water Science.

[32] Cohen Y., Lin N., Varin K.J., Chien D., Hicks R.F. (2013). Membrane Surface Nanostructuring with Terminally Anchored Polymer Chains. Functional Nanostructured Materials and Membranes for Water Treatment.

[33] Yang Z., Guo H., Tang C.Y. (2019). The upper bound of thin-film composite (TFC) polyamide membranes for desalination. J. Membr. Sci..

[34] Chen Y., Kim S., Cohen Y. (2021). Tuning the hydraulic permeability and molecular weight cutoff (MWCO) of surface nano-structured ultrafiltration membranes. J. Membr. Sci..

[35] Rahardianto A., McCool B.C., Cohen Y. (2008). Reverse Osmosis Desalting of Inland Brackish Water of High Gypsum Scaling Propensity: Kinetics and Mitigation of Membrane Mineral Scaling. Environ. Sci. Technol..

[36] Gilron J., Hasson D. (1987). Calcium sulphate fouling of reverse osmosis membranes: Flux decline mechanism. Chem. Eng. Sci..

[37] le Gouellec Y.A., Elimelech M. (2002). Calcium sulfate (gypsum) scaling in nanofiltration of agricultural drainage water. J. Membr. Sci..

[38] Shih W.-Y., Rahardianto A., Lee R.-W., Cohen Y. (2005). Morphometric characterization of calcium sulfate dihydrate (gypsum) scale on reverse osmosis membranes. J. Membr. Sci..

[39] Lee S., Lee C.-H. (2000). Effect of operating conditions on CaSO_4_ scale formation mechanism in nanofiltration for water softening. Water Res..

[40] Supekar O.D., Park D.J., Greenberg A.R., Gopinath J.T., Bright V.M. (2019). Real-time detection of early-stage calcium sulfate and calcium carbonate scaling using Raman spectroscopy. J. Membr. Sci..

[41] Thompson J., Lin N., Lyster E., Arbel R., Knoell T., Gilron J., Cohen Y. (2012). RO membrane mineral scaling in the presence of a biofilm. J. Membr. Sci..

[42] Lyster E., Cohen Y. (2007). Numerical study of concentration polarization in a rectangular reverse osmosis membrane channel: Permeate flux variation and hydrodynamic end effects. J. Membr. Sci..

[43] Lyster E., Au J., Rallo R., Giralt F., Cohen Y. (2009). Coupled 3-D hydrodynamics and mass transfer analysis of mineral scaling-induced flux decline in a laboratory plate-and-frame reverse osmosis membrane module. J. Membr. Sci..

[44] McCool B.C., Rahardianto A., Faria J., Kovac K., Lara D., Cohen Y. (2010). Feasibility of reverse osmosis desalination of brackish agricultural drainage water in the San Joaquin Valley. Desalination.

[45] Cohen Y., Rahardianto A., Christofides P.D., Thompson J.F., Gao L. (2016). Pilot-Scale Evaluation of High Recovery Desalination of Agricultural Drainage Water with Smart Integrated Membrane Systems (SIMS). Desalination and Water Purification Research and Development Program Final Report No. 173.

[46] Varin K.J., Lin N.H., Cohen Y. (2013). Biofouling and cleaning effectiveness of surface nanostructured reverse osmosis membranes. J. Membr. Sci..

[47] Uchymiak M., Rahardianto A., Lyster E., Glater J., Cohen Y. (2007). A novel RO ex situ scale observation detector (EXSOD) for mineral scale characterization and early detection. J. Membr. Sci..

[48] Klimchouk A. (1996). The dissolution and conversion of gypsum and anhydrite. Int. J. Speleol..

[49] Zhang X., Tian J., Gao S., Shi W., Zhang Z., Cui F., Zhang S., Guo S., Yang X., Xie H. (2017). Surface functionalization of TFC FO membranes with zwitterionic polymers: Improvement of antifouling and salt-responsive cleaning properties. J. Membr. Sci..

[50] Darvishmanesh S., Qian X., Wickramasinghe S.R. (2015). Responsive membranes for advanced separations. Curr. Opin. Chem. Eng..

[51] Drechsler A., Elmahdy M.M., Uhlmann P., Stamm M. (2018). pH and Salt Response of Mixed Brushes Made of Oppositely Charged Polyelectrolytes Studied by in Situ AFM Force Measurements and Imaging. Langmuir.

[52] Meng J., Cao Z., Ni L., Zhang Y., Wang X., Zhang X., Liu E. (2014). A novel salt-responsive TFC RO membrane having superior antifouling and easy-cleaning properties. J. Membr. Sci..

[53] You M., Wang P., Xu M., Yuan T., Meng J. (2016). Fouling resistance and cleaning efficiency of stimuli-responsive reverse osmosis (RO) membranes. Polymer.

[54] Liu H., Yang S., Liu Y., Miao M., Zhao Y., Sotto A., Gao C., Shen J. (2019). Fabricating a pH-responsive membrane through interfacial in-situ assembly of microgels for water gating and self-cleaning. J. Membr. Sci..

[55] Sun C., Feng X. (2017). Enhancing the performance of PVDF membranes by hydrophilic surface modification via amine treatment. Sep. Purif. Technol..

[56] Israelachvili J.N. (2015). Intermolecular and Surface Forces.

[57] Feast W.J., Munro H.S. (1987). Polymer Surfaces and Interfaces.

[58] Marshall A., Munro P., Trägårdh G. (1993). The effect of protein fouling in microfiltration and ultrafiltration on permeate flux, protein retention and selectivity: A literature review. Desalination.

[59] Fane A., Fell C., Suki A. (1983). The effect of pH and ionic environment on the ultrafiltration of protein solutions with retentive membranes. J. Membr. Sci..

[60] Larson T.E., Buswell A.M., Ludwig H.F., Langelier W. (1942). Calcium carbonate saturation index and alkalinity interpretations [with discussion]. J. Am. Water Work. Assoc..

[61] Warsinger D.M., Swaminathan J., Guillen-Burrieza E., Arafat H.A., Lienhard V J.H. (2015). Scaling and fouling in membrane distillation for desalination applications: A review. Desalination.

[62] Wang Y.-N., Järvelä E., Wei J., Zhang M., Kyllönen H., Wang R., Tang C.Y. (2016). Gypsum scaling and membrane integrity of osmotically driven membranes: The effect of membrane materials and operating conditions. Desalination.

[63] Peña N., Gallego S., Del Vigo F., Chesters S. (2013). Evaluating impact of fouling on reverse osmosis membranes performance. Desalination Water Treat..

[64] Sarker N., Cherukupally P., Gourevich I., Wilbur J., Jons S., Bilton A. (2022). Multi-scale visualization of incipient CaCO_3_ scaling on the polyamide layer of reverse osmosis membranes. Desalination.

[65] Benecke J., Haas M., Baur F., Ernst M. (2018). Investigating the development and reproducibility of heterogeneous gypsum scaling on reverse osmosis membranes using real-time membrane surface imaging. Desalination.

[66] Tzotzi C., Pahiadaki T., Yiantsios S., Karabelas A., Andritsos N. (2007). A study of CaCO_3_ scale formation and inhibition in RO and NF membrane processes. J. Membr. Sci..

[67] Shmulevsky M., Li X., Shemer H., Hasson D., Semiat R. (2017). Analysis of the onset of calcium sulfate scaling on RO membranes. J. Membr. Sci..

